# Implications of population-level immunity for the emergence of artemisinin-resistant malaria: a mathematical model

**DOI:** 10.1186/s12936-018-2418-y

**Published:** 2018-08-02

**Authors:** Nick Scott, Ricardo Ataide, David P. Wilson, Margaret Hellard, Ric N. Price, Julie A. Simpson, Freya J. I. Fowkes

**Affiliations:** 1 Disease Elimination Program, Burnet Institute, Melbourne, VIC 3004 Australia; 2 Department of Epidemiology and Preventive Medicine, Monash University, Melbourne, VIC 3004 Australia; 3 Department of Infectious Diseases, Alfred Hospital, Melbourne, VIC 3004 Australia; 4 Global and Tropical Health Division, Menzies School of Health Research, Charles Darwin University, Darwin, NT Australia; 5 Centre for Tropical Medicine and Global Health, Nuffield Department of Clinical Medicine, University of Oxford, Oxford, UK; 6 Centre for Epidemiology and Biostatistics, Melbourne School of Population and Global Health, The University of Melbourne, Melbourne, VIC 3010 Australia; 7 Department of Infectious Diseases, Monash University, Melbourne, VIC 3004 Australia

**Keywords:** Africa, Malaria, Artemisinin, Drug resistance, Immunity, Mathematical model

## Abstract

**Background:**

Artemisinin-resistant *Plasmodium falciparum* has emerged in the Greater Mekong Subregion, an area of relatively low transmission, but has yet to be reported in Africa. A population-based mathematical model was used to investigate the relationship between *P. falciparum* prevalence, exposure-acquired immunity and time-to-emergence of artemisinin resistance. The possible implication for the emergence of resistance across Africa was assessed.

**Methods:**

The model included human and mosquito populations, two strains of malaria (“wild-type”, “mutant”), three levels of human exposure-acquired immunity (none, low, high) with two types of immunity for each level (sporozoite/liver stage immunity and blood-stage/gametocyte immunity) and drug pressure based on per-capita treatment numbers.

**Results:**

The model predicted that artemisinin-resistant strains may circulate up to 10 years longer in high compared to low *P. falciparum* prevalence areas before resistance is confirmed. Decreased time-to-resistance in low prevalence areas was explained by low genetic diversity and immunity, which resulted in increased probability of selection and spread of artemisinin-resistant strains. Artemisinin resistance was estimated to be established by 2020 in areas of Africa with low (< 10%) *P. falciparum* prevalence, but not for 5 or 10 years later in moderate (10–25%) or high (> 25%) prevalence areas, respectively.

**Conclusions:**

Areas of low transmission and low immunity give rise to a more rapid expansion of artemisinin-resistant parasites, corroborating historical observations of anti-malarial resistance emergence. Populations where control strategies are in place that reduce malaria transmission, and hence immunity, may be prone to a rapid emergence and spread of artemisinin-resistant strains and thus should be carefully monitored.

**Electronic supplementary material:**

The online version of this article (10.1186/s12936-018-2418-y) contains supplementary material, which is available to authorized users.

## Background

Artemisinin-based combination therapy (ACT) has been recommended by the World Health Organization (WHO) as the first-line treatment for *Plasmodium falciparum* malaria since 2006 [1], and over the last decade has contributed significantly to the major reductions (~ 40%) in the global burden of falciparum malaria [2]. The appearance of artemisinin-resistant falciparum malaria in the Greater Mekong Subregion from 2009 poses a significant public health threat [3–11] and raises concerns that resistance may emerge and become widespread in high-burden settings, such as Africa. Over 400 million doses of artemisinin are procured annually worldwide [2] and resistance to this crucial drug may reverse recent gains in the global burden of disease and ultimately lead to a rise in epidemics and malaria-attributable mortality.

Artemisinin-resistant *P. falciparum* malaria is primarily determined by single nucleotide polymorphisms in the *kelch13* (*k13*) gene [12, 13], which are associated with a suboptimal clinical response including parasite recrudescence and delayed parasite clearance. Parasite clearance can be quantified either by the detection of parasites in patients on day 3 of follow-up or by calculating the parasite clearance half-lives (PCt_½_) ≥ 5 h after a full course of artemisinin treatment [14]. The WHO defines areas to have ‘confirmed partial artemisinin resistance’ if ≥ 5% of patients carry *k13* mutations plus a slow clearing phenotype [15]. Whilst mutations in the *k13* gene have been reported in African isolates, they do not appear to be under strong selection [16] and clinically relevant artemisinin-resistant falciparum malaria is yet to be observed in Africa [17].

The situation is different in the Greater Mekong Subregion. Historically this region has been the epicentre for the emergence of multidrug resistant parasites, with resistance to chloroquine, sulfadoxine–pyrimethamine and mefloquine first appearing there over 25 years ago [18, 19]. In Cambodia in particular, the wide-spread availability of substandard anti-malarials and use of artemisinin monotherapy has resulted in extensive exposure to suboptimal doses of effective drug, enabling the selection of mutations conferring drug resistance [20, 21]. Evidence also suggests that other factors, such as transmission intensity, genetic diversity and host immunity, play important roles in reducing the emergence and spread of anti-malarial drug resistant parasites [22, 23]. Understanding the multifactorial nature of the drivers of drug resistance may help to explain why anti-malarial resistance has consistently arisen in the Greater Mekong Subregion and predict the potential emergence of resistance in Africa, which harbours the largest burden of *P. falciparum* malaria [24, 25].

Various modelling studies have attempted to quantify the positive associations between immunity and *P. falciparum* prevalence [26, 27] and immunity and parasite diversity [22] in different endemic settings. However, these factors have not been considered together in the context of the spread of drug resistance. There is a need to understand these factors in combination in order to make informed decisions relating to malaria control and elimination in different prevalence settings.

Here a population-based mathematical model was used, calibrated to data from the Greater Mekong Subregion, to expand on previous modelling work and establish a quantitative relationship between *P. falciparum* prevalence and the time from the introduction of strains that confer artemisinin-resistance (*k13* mutation plus a slow clearing phenotype, henceforth ‘mutants’) until the WHO criteria of confirmed partial artemisinin resistance is met. The model was used to investigate how this relationship is mediated by the prevalence and strength of various types of immunity and by the degree of selfing in the population, as measured by within-host mutation rates. By combining this population-level relationship with data from the Malaria Atlas Project [28, 29] (country-level prevalence estimates), the Worldwide Antimalarial Resistance Network [30] (resistance emergence calibration) and WHO World Malaria Reports [2] (treatment data for drug pressure calibration), the implications of these relationships on the heterogeneity of artemisinin resistance appearance across Africa were assessed.

## Methods

### Model description

**Fig. 1:**
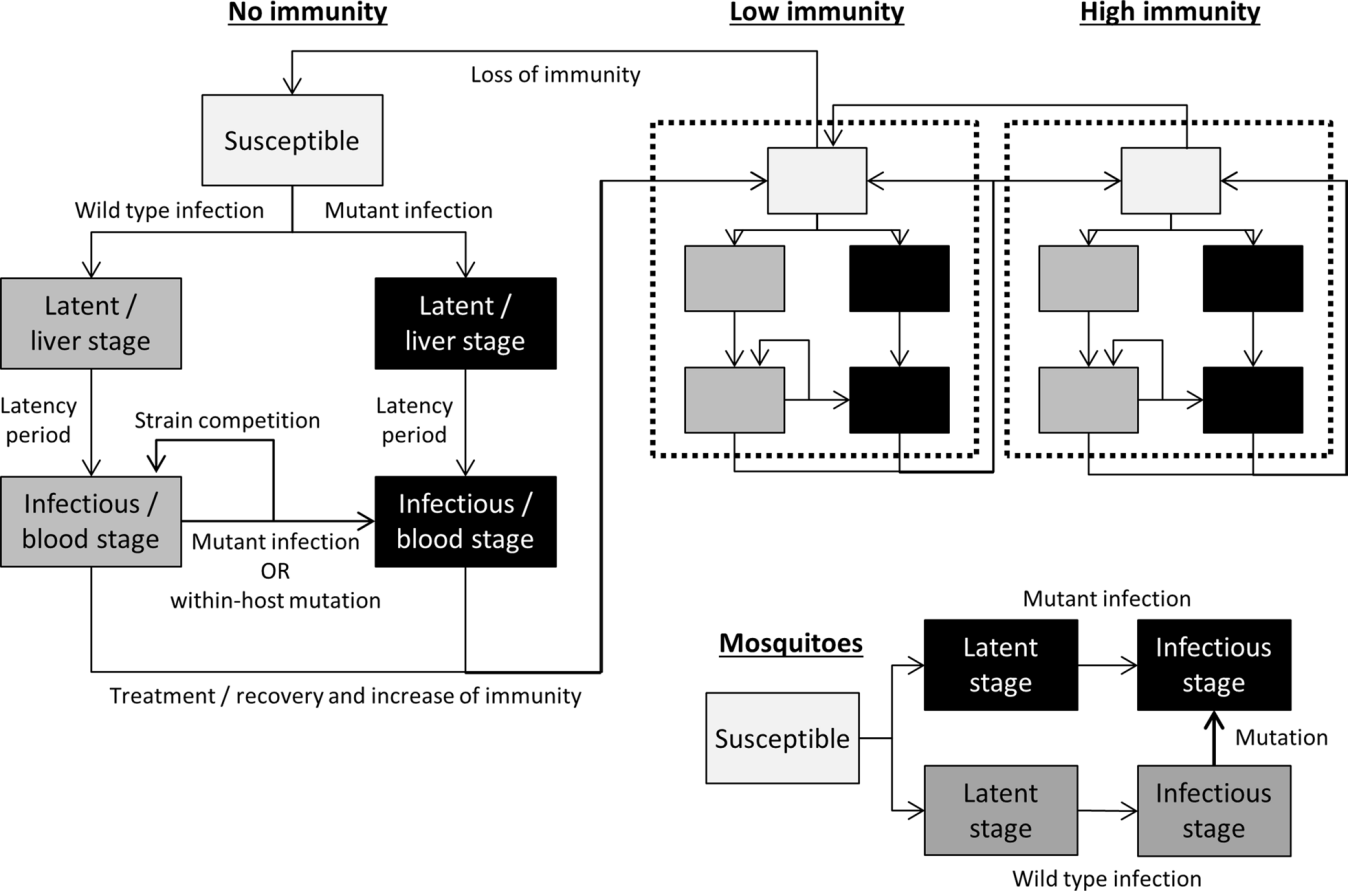
Model schematic. Individuals are either susceptible; infected with disease in the latent stage (approximating liver-stage infection); or infectious to mosquitoes (approximating gametocyte/blood-stage). Infection can occur with wild-type or mutant strains, and people with wild-type infections can become classified as having mutant infections either by being bitten by mutant-carrying mosquitoes (limited by strain competition) or by undergoing a within-host mutation process (limited by strain competition and enhanced by drug pressure). Exposure and recovery increases immunity level—lowering the risk of transmission from mosquito to human and from human to mosquito—and in the absence of re-infection immunity level reduces over time. Recovery and immunity are not modelled for mosquitoes

A population-based epidemiological mathematical model was developed to estimate the time taken for resistance to emerge in a malaria endemic population. The model simulated the transmission of two strains of malaria (“wild-type” or “mutant”—unless otherwise mentioned referring exclusively to *k13* mutations conferring the slow clearing phenotypes structure) within a human and a mosquito population. For the human population, the model included three levels of exposure-acquired immunity (none, low, high) and two types of immunity for each level (sporozoite/liver stage immunity and blood-stage/gametocyte immunity). A detailed model description is provided in the Additional file 1 and a model schematic is shown in Fig. 1.

People in the model who become infected with either “wild-type” or “mutant” strains of malaria experience a latent period (where they cannot infect mosquitoes) before becoming infectious and eventually recovering (either naturally clearing parasites or getting treated by drugs). After recovering, humans in the model gain exposure-acquired immunity, which lowers both their probability of developing infection following an infected mosquito bite (immunity at the sporozoite and liver stages) and the probability of a mosquito carrying an infection after biting an infectious human (blood-stage immunity).

People in the model who were susceptible could become infected with the mutant strain through direct transmission, while people who were already infected with wild-type strains could become classified as infected with the mutant strain through either direct transmission or to a lesser extent through a within-host “mutation” process. In both cases, the implication is that mutant parasites have appeared in the individual’s blood and multiplied to become a dominant strain of a co-infection. This is implicitly related to drug pressure as well as a relative fitness and recombination rate. Therefore, these processes were modelled to be constrained by a strain competition parameter (Fig. 1) and mutation rates were additionally modelled to be proportional to: the average number of ACT medicines administered per person per year (i.e. setting dependent), a recombination rate, and the dynamic proportion of infections in the population that were mutants. The mutation rate was also modelled to decrease as a person’s immunity increased, based on recent empirical data suggesting that people with greater exposure-acquired immunity are more efficient at suppressing the newly appearing strain [23]. Additional details, including model equations, are provided in the Additional file 1.

### Outcome measure

The outcome measure was “time to confirmed partial resistance”, defined as the time from mutant strain introduction (i.e. the time when the mutation feature in the model was turned on) until 5% of infectious humans were carrying *k13* mutant strains that confer a slow clearing phenotype. A curve plotting the time to resistance versus the prevalence of wild-type *P. falciparum* parasites (before the introduction of mutant strains) was developed by running the model independently for settings with varying prevalence of wild-type *P. falciparum* parasites.

### Calibration

A Particle Swarm Optimization Algorithm [31] was used in MATLAB to best fit the model’s mutation rates (human and mosquito populations), treatment parameters and strain competition parameters to observed data for settings in the Greater Mekong Subregion (Table 1). In each setting, the year that mutation started was approximated by the year that ACT was introduced.

**Table 1:** For various *P. falciparum* prevalence settings in the Greater Mekong Subregion, estimates of the time between artemisinin-based combination therapy (ACT) introduction and the setting being classified by the WHO as having confirmed partial resistance

Country	Year ACT introduced (estimated uncertainty)^a^	*P. falciparum* prevalence in year ACT introduced (95% confidence interval)^b^	Year when classified as area of confirmed partial resistance	Observed time to confirmed partial resistance (estimated uncertainty)^a^	Average number of ACTs administered per person per year^c^
Vietnam	1995 (1994–1996) [32, 33]	4% (3–5%) [29]	2009 [34]	14 years (13–15 years)	0.192
Thailand	1994 (1993–1995) [30, 32, 35]	7% (5–9%) [29]	2008 [34]	14 years (13–15 years)	0.031
Cambodia	2000 1999–2001) [30, 36]	6% (5–7%) [29]	2006 [34]	6 years (5–7 years)	0.996
Myanmar	2002 (2001–2003) [37]^d^	9% (8–10%) [29]	2008 [34]	6 years (5–7 years)	0.681
Lao PDR	2002 (2001–2003) [37]^d^	20% (17–22%) [29]	2013 [34]	11 years (10–12 years)	1.588

*Lao PDR* Lao People’s Democratic Republic

^a^ The precise year that ACT was introduced at scale is unclear, and so a ±1 year margin was used to capture this uncertainty

^b^ Prevalence was estimated by calculating the average of the results of all studies from the Malaria Atlas Project in the relevant years, with 95% confidence intervals estimated as two standard errors of the mean. Details are provided in Additional file 1: Table S4

^c^ Calculated from World Malaria Reports (treatment numbers) and UN Population Division data (population sizes). Details in Additional file 1: Table S3

^d^ The year the World Health Organization recommended the use of ACT in these settings

### Uncertainty analysis

A Monte Carlo uncertainty analysis was conducted to incorporate parameter uncertainties. The uncertainties of individual parameters were parametrized as independent probability distributions (Additional file 1: Tables S1, S2), and for settings with prevalence ranging from 0.1 to 70% (in 0.1% intervals), 100 simulations were undertaken using random, independent parameter draws. For each simulation the time to resistance was calculated and a density scatter plot was produced.

One-way sensitivity analyses were also undertaken to test the impact when the effects of each type of immunity were varied relative to the others; and the relative fitness of the mutant strain and mutation rates were varied.

### Estimating the time to resistance in Africa

To determine the implications of any relationships between prevalence, immunity and time-to-resistance, the model was used to estimate the percentage of people with *P. falciparum* infections who carried mutant strains across Africa in 2020, 2025 and 2030. Mutant strain introduction was estimated to occur in 2007 following the large scale-up of ACT across Africa [38, 39], and the modelled prevalence for each country was based on the prevalence of wild-type *P. falciparum* reported in the Malaria Atlas Project [28, 29] in 2007. Data on the prevalence of wild-type *P. falciparum* parasites was approximated as the prevalence among 2–10 year olds.

Within the host, the rate artemisinin resistant parasites emerge is expected to decrease linearly with the number of partner drugs co-administered [40]. Hence co-administration with a single partner drug would halve the rate at which artemisinin resistant parasites emerge relative to when artemisinin monotherapies are used. The model was calibrated to the Greater Mekong Subregion where the use of artemisinin monotherapies was common for many years prior to the WHO’s recommendation against their use in 2006 [41] and, therefore, when estimating the time-to-resistance in Africa (where the use of combination therapies has been more widespread) the rate of mutation in the model was divided by 1.5. Alternate scenarios where the rate of mutation in Africa was either the same or one half the rate calibrated to the Greater Mekong Subregion were used to derive plausible bounds for the estimates.

## Results

**Fig. 2:**
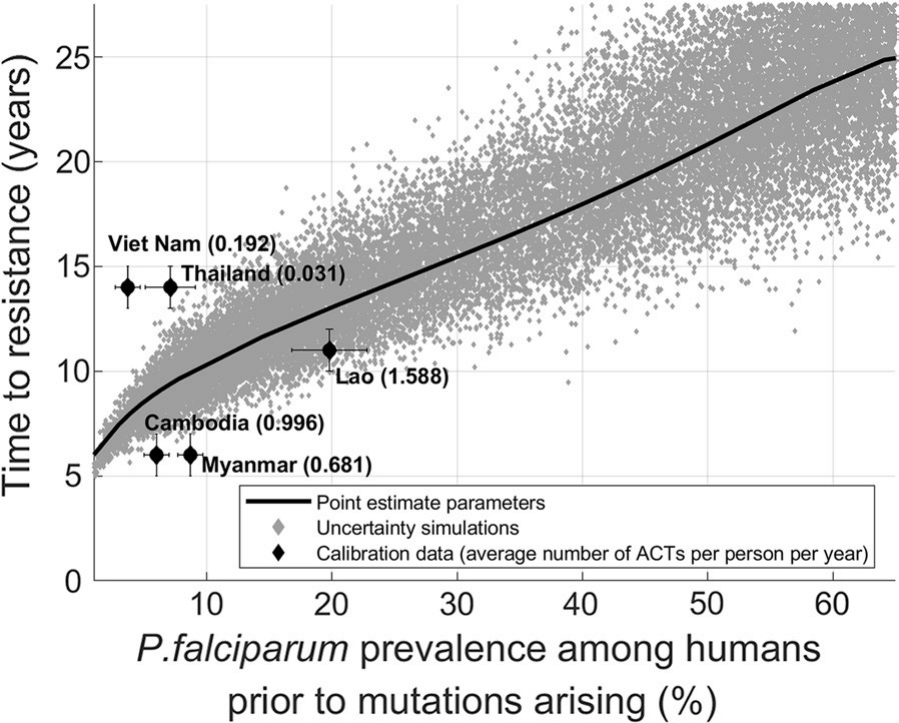
Time to confirmed partial artemisinin resistance (as measured by the time from mutant strain introduction until ≥ 5% of individuals carry parasites with K13 mutations and a slow clearing phenotype) in different *P. falciparum* prevalence settings, defined by the prevalence of wild-type *P. falciparum* infections in the human population in the year ACT was introduced. Using mean parameter estimates, the model predicts a longer time to detect drug resistant strains in areas with higher prevalence (solid black line). The results of the Monte Carlo uncertainty analysis are consistent with this finding (each dot represents a simulation using randomly drawn parameters). Calibration data points and their uncertainties (95% CIs) for Cambodia, Lao PDR, Myanmar, Thailand and Vietnam were obtained from the literature (MAP [29], WHO [34])

The resulting best-fit time to confirmed partial resistance versus *P. falciparum* prevalence curve is presented in Fig. 2. The model predicts that as *P. falciparum* prevalence increases, there will be a longer time interval before detection and classification of areas as having confirmed partial artemisinin resistance according to the WHO definition (Fig. 2, solid line). For example, in areas with a *P. falciparum* prevalence of 60%, the model estimates that it would take approximately 25 years from mutation appearance before the area is classified as having confirmed partial resistance, compared to only 10 years in an area with 10% prevalence. In particular, in some higher prevalence settings (> 30%) mutant strains may circulate for more than 10 years longer than in low prevalence settings (< 10%) before the areas are classified as having confirmed partial artemisinin resistance. The relationship between *P. falciparum* prevalence and time to confirmed partial resistance appears robust given parameter uncertainties (Fig. 2, grey scatter plot); however with increasing prevalence there is increasing uncertainty as to how long resistance will take to emerge.

### Mutation rates versus time to confirmed partial resistance

**Fig. 3:**
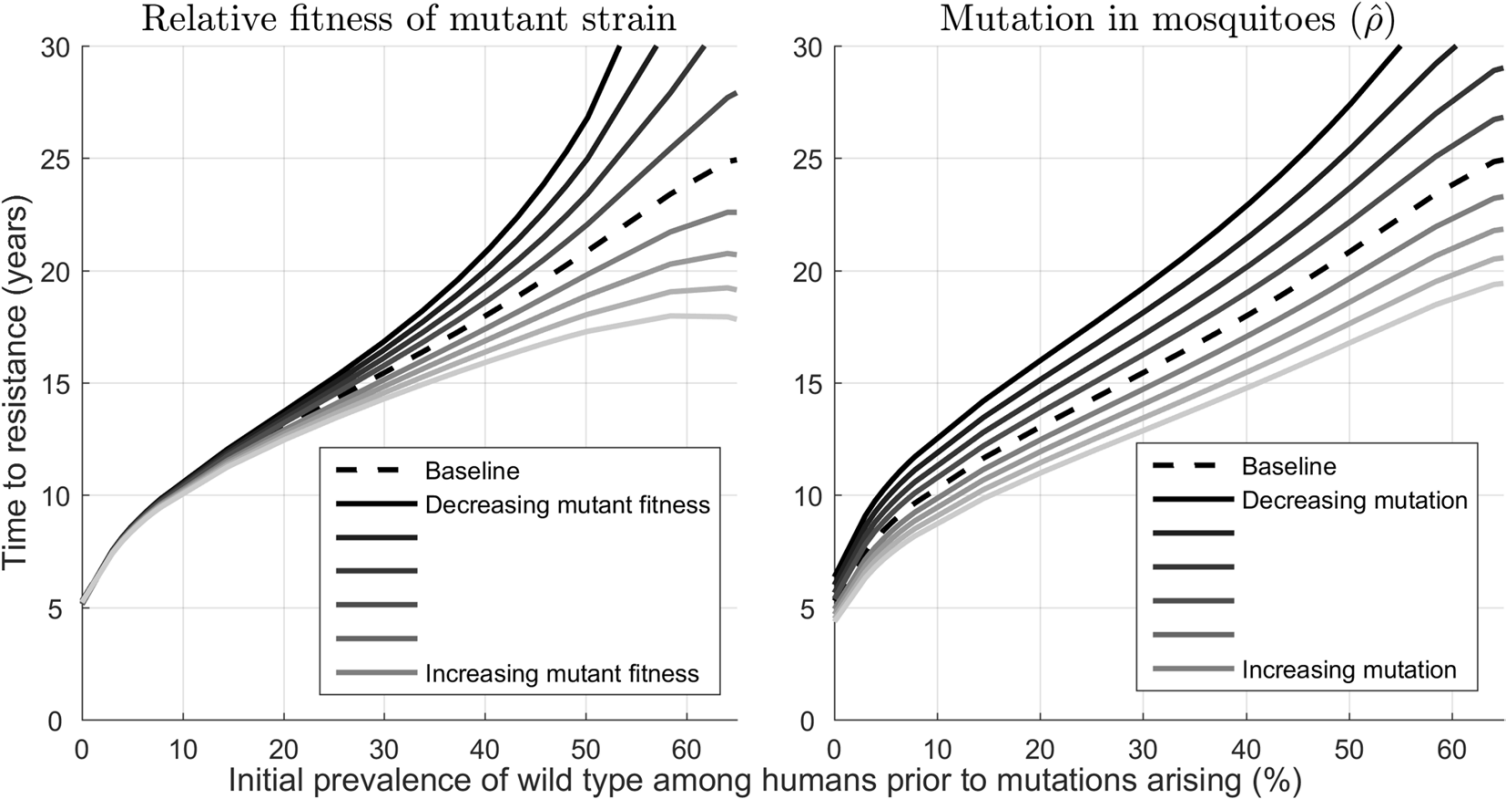
The effects of varying the relative fitness of the mutant strain and the mutation rate in the mosquito population on the time to confirmed partial resistance (as measured by the time from mutant strain introduction until ≥ 5% of patients carry parasites with K13 mutation and a slow-clearing phenotype). The relationship between prevalence and time to resistance classification as the relative fitness of the mutant strain is varied in 5% relative increments (left), and as the mutation rate is varied among the mosquito population in 5% relative increments (right)

When the mutation rate was varied in a sensitivity analysis, irrespective of its actual value, the time to resistance was always longer in areas of higher malaria prevalence (Fig. 3); however, there was uncertainty in the actual time that resistance might take to emerge. As might be expected, decreasing the relative fitness of the mutant strain or decreasing the mutation rate within mosquitoes delayed the time to resistance for a given initial prevalence. This indicates that determining the relative fitness and mutation rate will be crucial for accurate estimation of the emergence of resistance in particular settings.

### Immunity versus time to confirmed partial resistance

In the base scenario, immunity at the sporozoite and liver stages was assumed to reduce the probability of a human developing an infection following an infected mosquito bite by 25% [42] and 50% [42, 43] for individuals with low and high levels of immunity, respectively, while immunity at the blood-stage/gametocyte stage was assumed to lower the probability of a mosquito carrying an infection after biting an infectious human by 40% [44] and 80% [44] for individuals with low and high levels of immunity, respectively. In the absence of data to suggest otherwise, the base scenario assumed that the effects of immunity were equal for both wild-type and mutant strains.

**Fig. 4:**
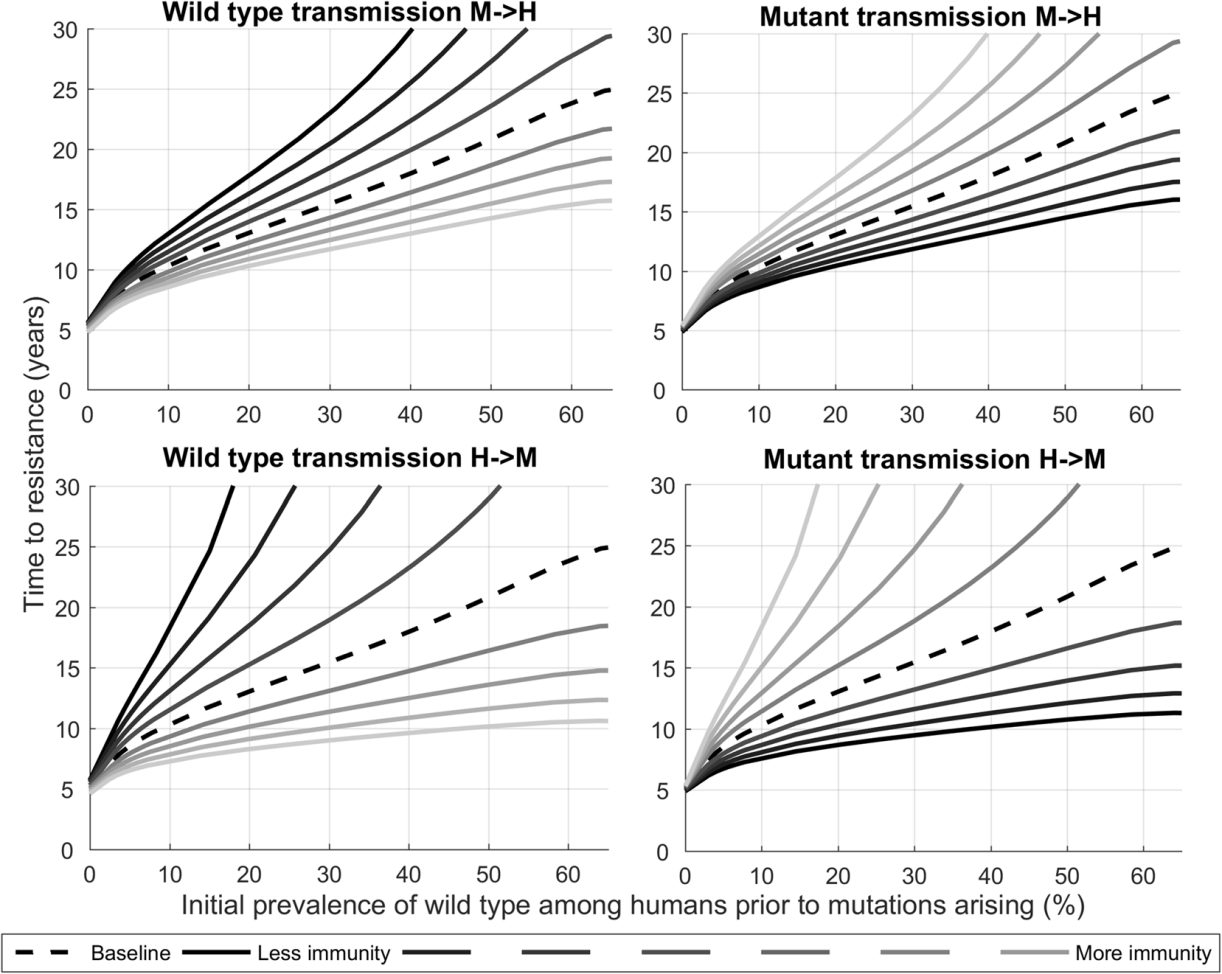
The effects of differing immunity on time to confirmed partial resistance (as measured by the time from mutant strain introduction until ≥ 5% of patients carry parasites with K13 mutation and a slow-clearing phenotype). The relationship between prevalence and time to confirmed partial resistance classification as the effects of immunity are increased/decreased in 1% relative increments for: wild-type transmission from mosquitoes to humans (top-left, from base values of 25%/50% for individuals with low/high immunity [42, 43]); mutant transmission from mosquitoes to humans (top-right, from base values of 25%/50% for individuals with low/high immunity [42, 43]); wild-type transmission from humans to mosquitoes (bottom-left, from base values of 40%/80% for individuals with low/high immunity [44]); and mutant transmission from humans to mosquitoes (bottom-right, from base values of 40%/80% for individuals with low/high immunity [44])

When the effects of sporozoite and blood-stage immunity were varied in a sensitivity analysis, irrespective of their actual effect sizes, the time to resistance was always longer in areas of higher malaria prevalence (Fig. 4); however, for a given prevalence the time to confirmed partial resistance was sensitive to changes in the effect size of immunity on wild-type strains relative to mutant strains. For example, immunity was assumed to have an equal effect on all strains; however, for a given prevalence, decreasing the effect of immunity on wild-type transmission from mosquitoes to humans by 4%—*for fixed mutant immunity effects*—could extend the time to resistance by more than 25% (Fig. 4, top-left). This is due to competitive advantage, since in this scenario wild-type strains were fitter and able to circulate more easily than mutant strains, which are inhibited by immunity relative to wild-type strains. Conversely, decreasing the effect of immunity on mutant transmission from mosquitoes to humans—*for fixed wild*-*type immunity effects*—gave mutants the competitive advantage, and decreased the time to resistance (Fig. 4, top-right). These effects were even more pronounced for immunity at the blood-stage (transmission from humans to mosquitoes; Fig. 4, bottom).

### Time to confirmed partial resistance in Africa

**Fig. 5:**
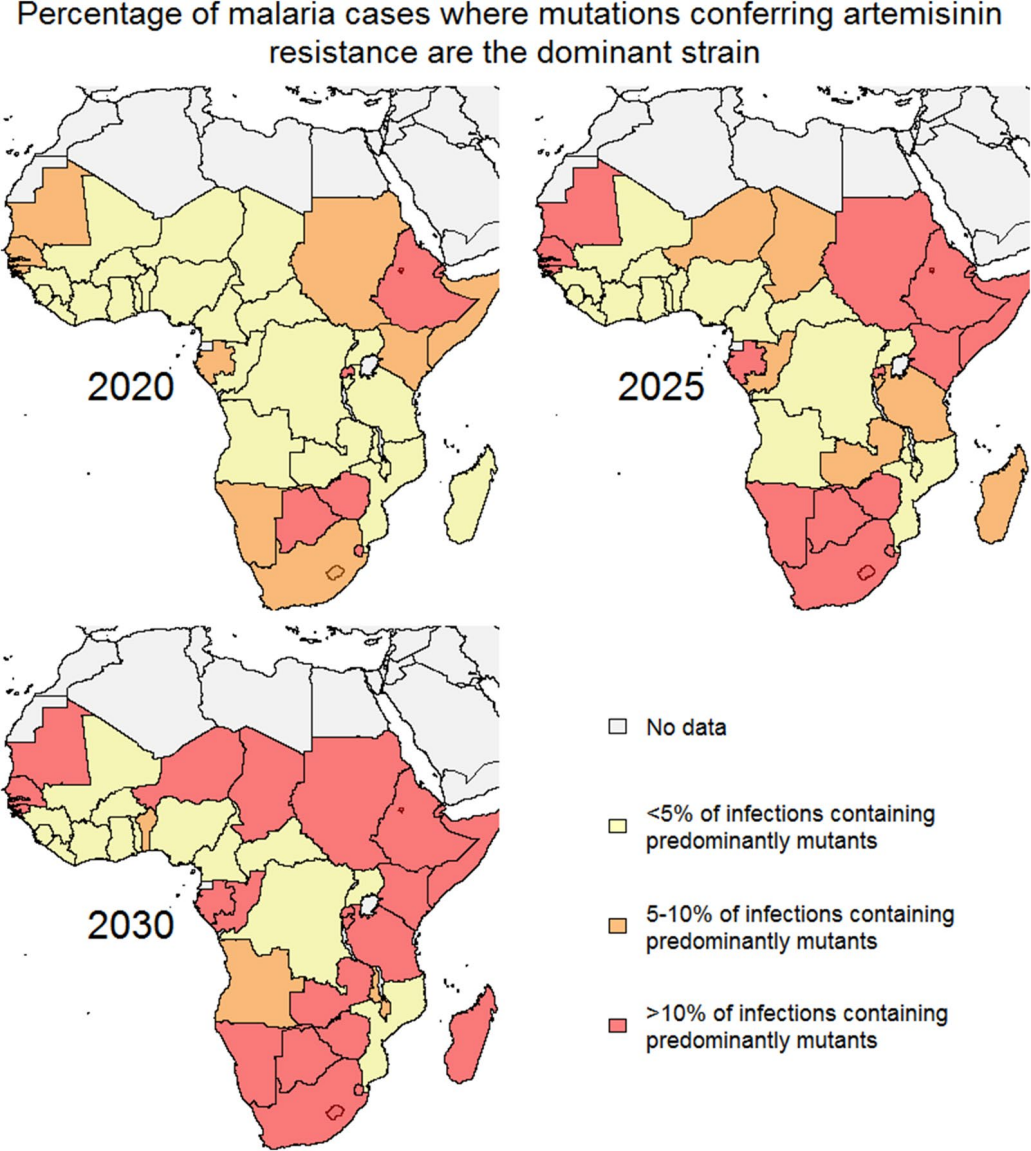
Projected emergence of artemisinin resistance in Africa. The estimated percentage of *P. falciparum* infections in African countries that contain ‘mutants’ (K13 mutations conferring the slow clearing phenotypes) as the most prevalent within-host strain; 2020, 2025 and 2030. Estimates are based on the prevalence of malaria reported in the Malaria Atlas Project [28, 29] in 2007 before the large scale-up of artemisinin-based combination therapy across Africa [38, 39]

The model suggests that if a similar relationship between prevalence, immunity and time-to-resistance were to hold in African settings, there would be considerable heterogeneity in the emergence of artemisinin resistance in the region. When the relationship derived from the Greater Mekong Subregion was applied to Africa, even by 2020 many countries with a lower prevalence (< 10%) at the time of ACT introduction, (Botswana, Eritrea, Ethiopia, Gabon, Gambia, Guinea-Bissau, Kenya, Mauritania, Namibia, Rwanda, Senegal, Somalia, South Africa, South Sudan, Sudan and Zimbabwe) were estimated to have enough *P. falciparum* parasites with *k13* mutations in circulation to meet the WHO classification of confirmed partial artemisinin resistance (Fig. 5). For areas with a prevalence between 10 and 25% at the time of ACT introduction (Burundi, Chad, Congo, Madagascar, Niger, United Republic of Tanzania and Zambia), artemisinin resistance was estimated to have emerged by 2025, and for areas with a prevalence between 25 and 37% at the time of ACT introduction (Angola, Benin, Malawi and Mozambique) artemisinin resistance was estimated to have emerged by 2030. For areas of higher prevalence at the time of ACT introduction (> 38%), such as Burkina Faso, Cameroon, Central African Republic, Côte d’Ivoire, Democratic Republic of the Congo, Equatorial Guinea, Ghana, Guinea, Liberia, Mali, Nigeria, Sierra Leone, Togo and Uganda, artemisinin resistance was estimated to take longer to emerge, with the model predicting that these areas are likely to meet the WHO resistance classification criteria by approximately 2040.

## Discussion

Understanding how and when anti-malarial resistant parasites emerge in a population is critical for prioritizing malaria control and elimination policies and optimizing treatment guidelines. Using a population-based mathematical model, this study demonstrated that in high prevalence settings, *P. falciparum* artemisinin-resistant strains may circulate for more than 10 years longer than in low prevalence settings before the areas are classified as having confirmed partial artemisinin resistance according to WHO criteria [15]. The derived relationships were robust to parameter uncertainties, in particular the relative magnitude of various types of immunity and within-host mutation rates (influenced by drug pressure and the relative fitness of the mutant strain). Furthermore, the relationships between prevalence and time-to-resistance predicted by the model imply considerable heterogeneity in the emergence of artemisinin-resistance across Africa, an important consideration as endemic countries move towards malaria elimination. These predictions presents a similar pattern and timeline to what was observed with the emergence of chloroquine resistance, which was introduced on large-scale in the early 1950s and led to confirmed chloroquine resistance in 1960 in the Cambodia–Thailand area but took almost 20 years longer to be confirmed in Africa [45].

The estimated decreased time to confirmed resistance in areas of low *P. falciparum* prevalence can be explained by low genetic diversity and low naturally acquired immunity. In areas of low *P. falciparum* prevalence, fewer individuals are co-infected with genetically different parasite strains, leading to a greater degree of selfing (the recombination of two genetically-identical gametes) and a lower degree of wild-type diversity [46–48]. As *P. falciparum* prevalence and immunity increases, clinically immune individuals can also act as an ecological reservoir for *P. falciparum* parasites (including infectious gametocytes), supporting onward transmission and parasite diversity. Within the model, settings with lower prevalence also had lower levels of immunity. This led to a greater overall within-host mutation rate, which facilitated the faster appearance of mutant strains that could spread more easily due to the lack of population-level immunity. These findings are consistent with observations that *k13* mutants are detected earlier in areas of the Greater Mekong Subregion with lower compared to higher *P. falciparum* prevalence, as well as global observations of anti-malarial resistance repeatedly emerging in the Greater Mekong Subregion, an area of relatively low malaria prevalence [10, 17, 49].

The model predicted that despite the slower spread of resistant parasites in areas of high prevalence, many areas of Africa may still reach the WHO confirmed partial resistance classification criteria by 2030. Moreover, these predictions may be overestimated, since they are based on the *P. falciparum* prevalence in African countries when artemisinin was introduced in 2007. Since 2007, successful malaria control programmes in many of these countries have reduced the prevalence, which the model suggests would also reduce the time to reach the WHO confirmed partial resistance classification criteria.

The findings of this study have significant implications for policy makers. Firstly, malaria control programmes typically focus on transmission reduction activities in areas with the highest prevalence, but results presented here suggest that elimination activities in areas of very low prevalence will be critical for containing the emergence of resistant parasites; a strategy now being pursued by WHO and National Malaria Control Programmes in the Greater Mekong Subregion [50]. These areas of low prevalence will need to be prioritized for programmes to delay the emergence of artemisinin drug resistance [51, 52], for example the strategic choice of partner drugs as part of ACT [53]. Modelling by others has found that strategies such as introducing multiple first-line therapies or cycling therapies could have substantial benefits in delaying the emergence of resistance [54]. Second, if mutant parasites were allowed to spread to high-burden areas such as Africa, this will shift elimination from an achievable goal to a considerably harder and more costly goal. Lubell et al. [55] have estimated that the spread of artemisinin resistance to sub-Saharan Africa could lead to an additional 125,000 deaths per year and US$1.5 billion in productivity losses, and Slater et al. [56] estimated that resistance in Africa at similar levels to those observed in Cambodia could result in an additional 78 million cases over a 5-year period. Third, in areas of high prevalence, the current WHO classification may be insufficient to identify the presence of artemisinin-resistant mutant strains, which in some cases may have been circulating for up to 10 years without detection. This has implications for migrants and travellers who may be exposed to artemisinin resistant mutant strains and risk carrying them to areas of low prevalence where they can spread more easily facilitating the global spread of artemisinin resistance.

This modelling study has a number of limitations. First, the year that resistance was predicted to emerge in Africa was found to depend on within-host mutation rates; however these are largely unknown at a population level and are influenced by genetic factors [57]. Therefore, application of the model to Africa was performed to highlight the possible heterogeneity across the region, and estimates for the actual year of emergence should be taken as approximate particularly due to within country transmission heterogeneity and uncertainty around *P. falciparum* prevalence estimates. Second, a compartmental model was used, which is limited by assumptions of homogeneity at both population and individual levels. More complex and computationally intensive models, such as agent-based models, could be used to address variations in individuals’ risk of infection and infectivity, as well as capturing in greater detail within-host parasite densities, mutation processes and drug-pressure. Third, the model assumed constant mosquito population densities and no differences in vectorial capacity (according to species) or seasonal changes in population size and prevalence. This means that in areas of seasonal transmission the time to resistance will have been overestimated. Fourth, immunity was approximated using discrete categories (none, low, high), when it is likely to be a continuum. Whilst inclusion of partial immunity is an advance on previous models that have used a dichotomous immune status, subtle changes in age-related transmission dynamics will have been missed [58–63]. Fifth, drug-pressure was modelled to be proportional to the per-capita number of treatments administered rather than the doses taken and drug concentrations achieved at the individual level. This approach is different to models that explicitly include within-host dynamics (for example Nguygen et al. [54]); however it was used as a method of including an average drug pressure at a population-level only and does not explicitly capture drug coverage or background partner drug resistance. These averaged effects of drug pressure were approximated to be weaker in African settings than in the Greater Mekong Subregion where lower quality artemisinin-based combinations have historically been used, but it is unclear how different these effects should be. When the sizes of these effects were tested in the sensitivity analysis, it was found that as treatment quality increased (a decrease in drug pressure, Fig. 3), the relationship between time to resistance and initial *P. falciparum* prevalence became even more distinct, with artemisinin-resistant strains circulating for even longer before areas were classified as having confirmed partial artemisinin resistance.

## Conclusions

The time from the emergence of artemisinin resistant malaria falciparum strains to the detection of confirmed partial artemisinin resistance is multifactorial and independently related to malaria prevalence, immunity and drug pressure/relative strain fitness within hosts. The model suggests that a more rapid expansion of artemisinin resistant parasites is likely in areas of low transmission and low immunity, which may explain why drug resistant malaria repeatedly arises in the Greater Mekong Subregion. These findings also suggest that in populations where control strategies are in place that reduce malaria transmission and hence immunity, artemisinin resistant strains may emerge and spread rapidly. Future models incorporating human within-host heterogeneity (including different mutation mechanisms) and heterogeneity in malaria transmission would allow fine-scale predictions of time to confirmed artemisinin resistance. However, these broad-scale conservative predictions are critical for strategic timelines of malaria control policies in areas where artemisinin resistance is yet to emerge.

## Additional file

**Additional file 1.** Supplementary material.

## Abbreviations

ACT: artemisinin-based combination therapy
K13: kelch13
MAP: Malaria Atlas Project
PCt½: parasite clearance half-life
WHO: World Health Organization
