# Supplementary material for: Implications of population-level immunity for the emergence of artemisinin-resistant malaria: a mathematical model
Source: Malar J. 2018 Aug 2;17:279. doi: 10.1186/s12936-018-2418-y (PMC6071336; doi:10.1186/s12936-018-2418-y)
Supplement: Supplementary file 1 — Additional file 1. Supplementary material. [file 12936_2018_2418_MOESM1_ESM.docx]

**Additional File 1: Supplementary material**

**Detailed model description**

We used compartmental models of malaria transmission among humans and mosquitoes, linked through their dynamic infection parameters, as shown in Figure S1, with parameters as described below.


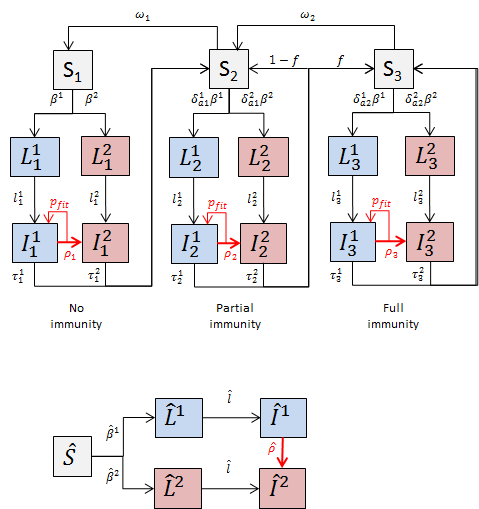


Figure S1: Model schematic: Individuals are either susceptible (S); infected with disease in the latent stage (L—approximating liver-stage infection); or infectious to mosquitoes (I—approximating gametocytes blood-stage). Subscripts represent different levels of immunity (1=none, 2=low, 3=high) and superscripts represent parasite strain (1=wild-type, 2=K13 mutant strain).

*Compartments*

People in the model were distinguished as either: susceptible (S); infected with disease in the latent stage (L—approximating liver-stage infection) or infectious to mosquitoes (I—approximating presence of circulating gametocytes). People in the latent and infectious stages were further classified as carrying either a wild-type strain (superscript 1) or a *kelch13* mutant strain that confers a slow clearance phenotype (hereafter “mutant strain”, superscript 2). For ease of communication, individuals co-infected with wild-type and K13 mutant strains were classified according to their dominant strain; however people who were classified as being infected with a wild-type strain could become classified as being infected with a mutant strain by two mechanisms: being bitten and infected by a mosquito carrying the mutant strain, or through a within-host mutation rate ρ (both discussed below).

*Dynamics*

When the model was run, susceptible individuals became infected and moved to the latent compartment at rates $\beta^{i}$ (for strain $i$) that were proportional to: the number of mosquito bites per month N; the per-bite probability of an infected mosquito transmitting sporozoites; and the proportion of mosquitoes that were currently infected with strain $i$. After an average duration of $1/l$ months in the latent stage, individuals progressed to the infectious stage, where they spent an average duration 1/τ months before infection was cleared. A proportion $\eta$ of clearance was due to treatment, compared to $(1-\eta)$ as a result of natural suppression. Treatment efficacy $\epsilon$ could be varied depending on the setting and types of drugs administered as part of the artemisinin combination therapy. In particular, treatment efficacy was lowered (from 95% [1] to 90%) for the mutant strain, due to the increased proportion of treatment failures. 90% was chosen as the lower efficacy as the WHO recommends that failure rates greater than 10% should trigger a change in malaria treatment policy [2]. By implementing this difference between strains, the model implicitly produces slightly longer clearance times for the mutant strain, as additional individuals who do not clear following treatment are modelled to remain in the infectious pool while they undertake a second treatment course.

*Co-infection and relative fitness*

Individuals who were classified as infected with the wild-type strain could become infected with the mutant strain through direct transmission at the same rate $\beta^{2}$, however only a proportion $p_{fit}$ (representing the relative fitness of the mutant strain) of these co-infected individuals became classified as infected with mutant strains, with the remaining proportion $(1-p_{fit})$ retaining only wild-type infections.

*Immunity*

The model includes three levels of immunity (none, low, high) and two types of immunity for each level: immunity at the sporozoite and liver stages (lowering the probability of a human developing infection following an infected mosquito bite) and blood-stage immunity (lowering the probability of a mosquito carrying an infection after biting an infectious human). The former is effectively modelling within-host resistance to sporozoites entering the liver, while the latter is effectively modelling a lowered gametocyte density among humans with immunity. In addition, although the base parameters consider immunity to affect both wild-type and mutant strains equally, a sensitivity analysis was conducted to test the effects if this were not the case. In reality the level of immunity is more likely to be a continuous rather than a categorical variable; however, by considering three levels of immunity we have expanded on previous population-based models that consider immunity dichotomously [3-8].

Specifically, following clearance of infection, “low immunity” was obtained (subscript 2), which lasted for an average duration 1/ω_1_ months. Low immunity was assumed to affect both the likelihood of sporozoite transmission from mosquito to human, and gametocyte transmission from human to mosquito: people with low immunity had wild-type and K13 mutant infection rates scaled by factors of $\delta_{a1}^{1}$ and $\delta_{a1}^{2}$ respectively, and were less infectious to mosquitoes by factors of $\delta_{b1}^{1}$ and $\delta_{b1}^{2}$ respectively ($\delta_{a1}^{1},\delta_{a1,}^{2}\delta_{b1}^{1},\delta_{b1,}^{2}$<1). If people with low immunity were re-infected and again cleared their infection, they either obtained “high immunity” (subscript 3) with probability $f$, or retained low immunity for a further 1/ω_1_ months. High immunity lasted for an average duration 1/ω_2_ and affected transmission analogously to low immunity—transmission rates were scaled similarly by factors $\delta_{a2}^{1},\delta_{a2}^{2},\delta_{b2}^{1}$ and $\delta_{b2}^{2}$ (where $\delta_{a2}^{i}<\delta_{a1}^{i}<1$, and $\delta_{b2}^{i}<\delta_{b1}^{i}<1$ for $i=1,2$). Further infection and clearance among people with high immunity extended the duration by an additional 1/ω_2_ months.

*Mosquitoes*

Dynamics in the mosquito model were identical to the human model except that:

- no recovery was modelled (and hence no immunity), since the mosquito lifecycle was assumed to be shorter than the infection clearance time;
- the force of infection was proportional to the probability of an infectious *human* transmitting gametocytes in a single bite, and the immunity weighted infectious human population; and
- mosquitoes could not become co-infected with strains.

*Mutation*

As this is a population-based model, we have used a single parameter to represent the “rate of mutation” (ρ). Within the host, mutation is a complex, multi-step process which should be considered for within-host models; however, for population-based models it is common practice and satisfactory to simplify this to a single parameter [3, 4, 9]. In order for a person to move from an $I^{1}$ (wild-type infected) compartment to an $I^{2}$ (mutant infected) compartment, mutations in the circulating parasite population must not only arise in the individual’s blood, but also multiply to become a dominant strain (i.e. the dominant strain of a co-infection). This is implicitly related to drug pressure as well as a relative fitness and recombination rate, both of which depend on the multiplicity of infections in the setting being modelled. Therefore these processes were dampened by a strain competition parameter $p_{fit}$, and mutation rates $\rho$ were modelled to be setting dependent and proportional to: a base mutation rate, the average number of ACTs administered per person per year and the proportion of mutant infections in the population (i.e. ${\sum_{k=1,2,3} I_{k}^{2}}/{\sum_{k=1,2,3} \left( I_{k}^{1}+I_{k}^{2} \right)}$ for subscripts 1, 2 and 3 representing people with no, low and high immunity respectively). Further, *ρ* will decrease as a person’s immunity increases (i.e. $\rho_{1}>\rho_{2}>\rho_{3}$) and their immune system becomes more efficient at supressing the newly appearing strain [10].

For mosquitoes, $\hat{\rho}$ is proportional to: a mutation rate and the current proportion of mutant infections in the population i.e. $\hat{I}^{2}/{(\hat{I}}^{1}+\hat{I}^{2}$). Selective pressure being exerted by drugs is not included as part of the mutations in the mosquito population. In a sensitivity analysis we examined the effects of varying the mutation rate, representing variations in the genetic diversity of different settings. The specific constants used were calibrated to data from the Greater Mekong Subregion (Table 1), using the method described in the calibration section below.

*Calibration*

Data from settings in the Greater Mekong Subregion (Table 1) was used to calibrate the model’s base mutation rates for human ($\rho_{1},\rho_{2}, \rho_{3})$ and mosquito populations $(\hat{\rho}$), the proportion of clearance due to treatment for each immunity level ($\eta_{1}, \eta_{2}, \eta_{3}$) and the strain competition parameter ($p_{fit}$). This was done as a two-step process. The first used a Particle Swarm Optimization (PSO) algorithm [11] in MATLAB to estimate the biting rates that produced the prevalence in each of the calibration settings, plus three global parameters (independent of prevalence) for the proportion of clearance due to treatment for each immunity level ($\eta_{1}, \eta_{2}, \eta_{3}$) that would best produce the treatment numbers in each setting (i.e. five independent biting rates for the five settings plus three treatment clearance parameters – the objective function ran the model for all settings and optimized the combined fit). This was done in the absence of any mutant strains. The second calibration procedure used another PSO algorithm to estimate global parameters (independent of prevalence) for the model’s mutation rates among humans (for each level of immunity:$\rho_{1},\rho_{2}, \rho_{3}$) and mosquitos ($\hat{\rho}$), and the strain competition parameter ($p_{fit}$) that best fit all of the observed time-to-resistance data points. In each setting, the year that mutation started was approximated by the year that artemisinin combination therapy was introduced. The assumption that treatment clearance and mutation parameters were global was made in order to assume no a-priori relationship between prevalence and resistance emergence, other than through drug pressure and immunity. Explicitly this assumption is that (1) individuals with the same level of immunity and same exposure to drugs will have the same mutation rate, irrespective of their setting; and (2) individuals with the same level of immunity have the same probability of naturally clearing parasites, irrespective of their setting. It is possible, for example, that people in lower prevalence settings may have faster mutation rates due to a number of other epidemiological factors; however this would make the actual relationship between prevalence and resistance emergence even more prominent than we have modelled.

The final values of $\eta_{1}, \eta_{2}, \eta_{3}, \rho_{1},\rho_{2}, \rho_{3}, \hat{\rho}$ and $p_{fit}$ from this process were used for the rest of the modelling; however the value of N was varied in order to simulate different prevalence settings.

*Outcome measures*

We used “time to confirmed partial artemisinin resistance” as the outcome measure, defined as the time from K13 mutant strain introduction (i.e. the time when the mutation feature in the model was turned on) until 5% of infectious humans were carrying K13 mutations that confer a slow clearing phenotype [12]. A time to confirmed partial artemisinin resistance versus initial wild-type prevalence curve was developed by running the model independently for settings with varying initial wild-type prevalence.

*Uncertainty analysis*

A Monte Carlo uncertainty analysis was conducted to incorporate parameter uncertainties. The uncertainties of individual parameters were parametrised as independent probability distributions as shown in Table S1 and Table S2, and for a settings with wild-type prevalence ranging from 0.1% to 70% (in 0.1% intervals), 100 simulations were undertaken using random, independent parameter draws. For each simulation the time to resistance was calculated and a density scatter plot was produced.

One-way sensitivity analyses were also undertaken to test the impact when the effects of each type of immunity were varied relative to the others; and mutation rates were varied among human and among mosquito populations.

*Prevalence range*

Calibrating the model to low prevalence areas in the Greater Mekong Subregion required the prevalence among the mosquito population to be greater than among the human population to prevent the epidemic from dying out at equilibrium (Figure S2). This meant that as the model was extrapolated to settings with extremely high (>70%) prevalence among the human population, the mosquito population reached a saturation level. Results have therefore not been generated for human populations with prevalence above 65% since (1) the population-level model used is likely to require parameter re-calibration to be used in these settings; and (2) the highest prevalence setting in Africa that we have applied the model to was below this (Côte d'Ivoire, 64%).

*Estimating the time to resistance in Africa*

We used the modelled relationship between initial wild-type prevalence and time to resistance to estimate when countries in Africa might expect circulating mutant parasite prevalence to reach the WHO classification of confirmed partial resistance. Initial wild-type prevalence data was not available for the entire population of countries, and so was approximated as the prevalence among 2-10 year olds as reported in the Malaria Atlas Project [13, 14]. Mutant strains introduction was estimated to be in 2007, following the large scale up of artemisinin combination therapy across Africa [15, 16].

Within the host, the rate artemisinin resistant parasites emerge is expected to decrease linearly with the number of partner therapies co-administered [17]. For example, the co-administration of a partner drug would halve the rate artemisinin resistant parasites emerge relative to when artemisinin monotherapies are used. Our model was calibrated to the Greater Mekong Subregion where the use of artemisinin monotherapies was common for many years prior to the WHO’s ban on monotherapies in 2006 [18], and therefore when estimating the time-to-resistance in Africa (where the use of combination therapies has been more widespread) the rate of mutation in the model was divided by 1.5. Alternate scenarios where the rate was either the same or one half the rate calibrated to the Greater Mekong Subregion were used to derive error margins for our estimates.

**Parameter values**

Table S1: Parameters and their uncertainties for the human model.

|  | | | **HUMANS** | | | | |
| --- | --- | --- | --- | --- | --- | --- | --- |
| *Symbol* | | *Description* | | *Strain* | *Value* | *Range; distribution* | *Citation* |
| *Population* | | | | | | | |
| $pop$ | Size of human population | | |  | 100 |  | Arbitrary |
| $\mu$ | Birth / death rate [1/average life expectancy] | | |  | 60 years^-1 | Normal distribution (mean=60 years, SD=5) | Assumed |
| *1/average duration of latency [time from bite to infectious]* | | | | | | | |
| $l_{1}^{1}$ | No immunity | | | Wild | 3.0438 months^-1  (i.e. 9.9 days) | 9-10 days; uniform distribution | [7, 19] for estimated value, [20] for range |
| $l_{2}^{1}$ | Low immunity | | | Wild | “ | “ | Assumed same as wild-type |
| $l_{3}^{1}$ | High immunity | | | Wild | “ | “ | “ |
| $l_{1}^{2}$ | No immunity | | | Mutant | “ | “ | “ |
| $l_{2}^{2}$ | Low immunity | | | Mutant | “ | “ | “ |
| $l_{3}^{2}$ | High immunity | | | Mutant | “ | “ | “ |
| *1/average duration of infectiousness* | | | | | | | |
| $\tau_{1}^{1}$ | No immunity | | | Wild | 4 months^-1  (i.e. 7.6 days) | 6-10 days; uniform distribution | Represents average time to seek treatment. From [21]: in a cohort study in western Cambodia 87% had cleared their *P. falciparum* infection within 1 month, and 13% remained parasitaemic for 2-4 months. A Poisson distribution was fit to these values to determine the average duration of infection (7.6 days) for people without immunity (given the study was in a low prevalence area – 2.2%). |
| $\tau_{2}^{1}$ | Low immunity | | | Wild | 0.5530 months^-1  (i.e. 55 days) | 44-66 days; uniform distribution | Estimated as half the time of people with full-immunity, to be consistent with the effect sizes of immunity levels. |
| $\tau_{3}^{1}$ | High immunity | | | Wild | 0.2765 months^-1  (i.e. 110 days) | 87-131 days; uniform distribution | [1, 22]; see supplement for sub-patent infection. |
| $\tau_{1}^{2}$ | No immunity | | | Mutant | 4 months^-1  (i.e. 7.6 days) | 6-10 days; uniform distribution | Assumed same as wild-type |
| $\tau_{2}^{2}$ | Low immunity | | | Mutant | 0.5530 months^-1  (i.e. 55 days) | 44-66 days; uniform distribution | Assumed same as wild-type |
| $\tau_{3}^{2}$ | High immunity | | | Mutant | 0.2765 months^-1  (i.e. 110 days) | 87-131 days; uniform distribution | Assumed same as wild-type |
| *Immunity* | | | | | | | |
| $\omega_{1}$ | Average duration of low immunity | | | Both | 1 year | Normal distribution (mean=1 years, SD=1 month) | Chitnis et al. [20] determine an immunity waning rate for a low-prevalence setting 2.7 x 10^-3^ days^-1^ |
| $\omega_{2}$ | Average duration of high immunity | | | Both | 5 years | Normal distribution (mean=5 years, SD=6 months) | Chitnis et al. [20] determine an immunity waning rate for a high-prevalence setting 5.5 x 10^-4^ days^-1^ |
| $f$ | Proportion who gain high immunity after reinfection with low immunity | | | Both | 0.1 | Beta distribution (variance = 0.25%) | Estimated |
| $\delta_{a1}^{1}$ | Reduction in M🡪H transmission with low immunity | | | Wild | 25% | Beta distribution (variance = 0.25%) | [23] |
| $\delta_{a2}^{1}$ | Reduction in M🡪H transmission with high immunity | | | Wild | 50% | Beta distribution (variance = 0.25%) | [23, 24] |
| $\delta_{a1}^{2}$ | Reduction in M🡪H transmission with low immunity | | | Mutant | 25% | Beta distribution (variance = 0.25%) | Assumed same as wild-type |
| $\delta_{a2}^{2}$ | Reduction in M🡪H transmission with high immunity | | | Mutant | 50% | Beta distribution (variance = 0.25%) | Assumed same as wild-type |
| $\delta_{b1}^{1}$ | Reduction in H🡪M transmission with low immunity | | | Wild | 40% | Beta distribution (variance = 0.25%) | [25] |
| $\delta_{b2}^{1}$ | Reduction in H🡪M transmission with high immunity | | | Wild | 80% | Beta distribution (variance = 0.25%) | [25] |
| $\delta_{b1}^{2}$ | Reduction in H🡪M transmission with low immunity | | | Mutant | 40% | Beta distribution (variance = 0.25%) | [25] |
| $\delta_{b2}^{2}$ | Reduction in H🡪M transmission with high immunity | | | Mutant | 80% | Beta distribution (variance = 0.25%) | [25] |
| *Rate of mutation in humans* | | | | | | | |
| $\rho_{1}$ | No immunity | | |  | Calibrated |  |  |
| $\rho_{2}$ | Low immunity | | |  | Calibrated |  |  |
| $\rho_{3}$ | High immunity | | |  | Calibrated |  |  |
| $p_{fit}$ | Relative fitness | | |  | Calibrated |  |  |
| *Transmission* | | | | | | | |
| N | Number of bites per month | | |  | Calibrated to setting prevalence | – |  |
| $\lambda_{MH}^{1}$ | Probability of transmission per bite, mosquito to human | | | Wild | 5% | 0.01-0.08 [20]; Beta distribution (variance = 0.01%) | [26] |
| $\lambda_{MH}^{2}$ | Probability of transmission per bite, mosquito to human | | | Mutant | 5% | Beta distribution (variance = 0.01%) | Assumed the same as wild-type |
| $\beta^{1}$ | Force of infection | | | Wild | $N\lambda_{MH}^{1}\times\frac{\hat{I}^{1}}{\# mosquitoes}$ | – | Model |
| $\beta^{2}$ | Force of infection | | | Mutant | $N\lambda_{MH}^{2}\times\frac{\hat{I}^{2}}{\# mosquitoes}$ | – | Model |

Table S2: Parameters and their uncertainties for the mosquito model.

| **MOSQUITOES** | | | | | |
| --- | --- | --- | --- | --- | --- |
| *Symbol* | *Description* | *Strain* | *Value* | *Range* | *Citation* |
| *Population* | | | | |  |
| $\hat{\mu}$ | Birth / death rate [1/average life expectancy] |  | 2.621 months^-1  (i.e. 11.6 days) | 8.8 – 16.5 days; uniform distribution | Varies between species but daily survival rates of mosquitoes in the wild have been found to be approximately constant with age, ranging between 0.77 and 0.87 [27]. Converting this to a mortality rate = -log(1-daily proportion that die); or to an average life span = 1/ rate. |
| *1/average duration of latency [time from bite to infectious]* | | | | | |
| $\hat{l}^{1}$ |  | Wild | 2.173 months^-1  (i.e. 14 days) | Normal distribution (mean=14 days, SD=2) [19, 28] | [29, 30] |
| $\hat{l}^{2}$ |  | Mutant | 2.173 months^-1  (i.e. 14 days) | Normal distribution (mean=14 days, SD=2) | Assumed the same as wild-type |
| *Rate of mutation* | | | | | |
| $\hat{\rho}$ |  | Wild | Calibrated |  |  |
| *Transmission* | | | | | |
| $\hat{N}$ | Average number of feeding occasions per mosquito per month (1/ time between feeds) |  | 9.5 months^-1  (i.e. every 3.2 days) | 2.7-3.8 days; uniform distribution | [31-33] |
| $\hat{pop}$ | Size of mosquito population |  | Calibrated relative to human population size based on N | ‑ | $pop*\frac{N}{\hat{N}}$ |
| $\lambda_{HM}^{1}$ | Probability of transmission per bite, human to mosquito | Wild | 0.47 | Beta distribution (variance = 0.0025) | [7, 20, 28, 34, 35] |
| $\lambda_{HM}^{2}$ | Probability of transmission per bite, human to mosquito | Mutant | 0.47 | Beta distribution (variance = 0.0025) | Assumed the same as wild-type |
| $I^{1}$ | Weighted prevalence of infectious humans | Wild | $\frac{I_{1}^{1}+\delta_{b1}^{1}I_{2}^{1}+\delta_{b1}^{1}I_{3}^{1}}{\# humans}$ | — | Model |
| $I^{2}$ | Weighted prevalence of infectious humans | Mutant | $\frac{I_{1}^{2}+\delta_{b1}^{2}I_{2}^{2}+\delta_{b1}^{2}I_{3}^{2}}{\# humans}$ | — | Model |
| $\hat{\beta}^{1}$ | Force of infection | Wild | $N\lambda_{HM}^{1}I^{1}$ | — | Model |
| $\hat{\beta}^{2}$ | Force of infection | Mutant | $N\lambda_{HM}^{2}I^{2}$ | — | Model |

Table S3: Treatment numbers and populations sizes used to calibrate drug pressure in the Greater Mekong Subregion.

|  | **Viet Nam** | **Thailand** | **Cambodia** | **Myanmar** | **Lao People's Democratic Republic** |
| --- | --- | --- | --- | --- | --- |
| Number of ACTs administered (World Malaria Reports) | | | | | |
| 2004 | 183,333 | 3,438 | 84,421 | missing | 16,200 |
| 2005 | 161,667 | 20,246 | 75,082 | 143,385 | 77,760 |
| 2006 | 231,508 | 34,848 | 112,495 | 392,085 | 140,640 |
| 2007 | 112,500 | 33,178 | 150,819 | 226,397 | 164,160 |
| 2008 | 109,725 | 26,150 | 81,090 | 187,102 | 287,160 |
| 2009 | 323,748 | 40,740 | 106,202 | 544,378 | 68,903 |
| 2010 | missing | 26,471 | 182,046 | 266,769 | 51,425 |
| 2011 | 110,576 | 5,642 | 120,529 | 569,607 | 56,340 |
| 2012 | 192,400 | 3,348 | 422,024 | 546,060 | 104,400 |
| 2013 | 141,570 | 15,069 | 117,547 | 371,663 | 48,470 |
| 2014 | 106,100 | 19,314 | 118,483 | 281,103 | 50,092 |
| Estimated population size (UN Population Division) | | | | | |
| 2004 | 83,420,166 | 65,229,843 | 13,095,627 | 49,521,721 | 5,664,585 |
| 2005 | 84,203,817 | 65,863,973 | 13,320,058 | 49,984,704 | 5,745,012 |
| 2006 | 85,034,609 | 66,029,583 | 13,528,764 | 50,334,366 | 5,848,118 |
| 2007 | 85,865,400 | 66,195,193 | 13,737,469 | 50,684,028 | 5,951,225 |
| 2008 | 86,696,192 | 66,360,804 | 13,946,175 | 51,033,689 | 6,054,331 |
| 2009 | 87,526,983 | 66,526,414 | 14,154,880 | 51,383,351 | 6,157,438 |
| 2010 | 88,357,775 | 66,692,024 | 14,363,586 | 51,733,013 | 6,260,544 |
| 2011 | 89,375,740 | 66,945,491 | 14,606,449 | 52,165,841 | 6,368,840 |
| 2012 | 90,393,705 | 67,198,958 | 14,849,311 | 52,598,669 | 6,477,136 |
| 2013 | 91,411,671 | 67,452,425 | 15,092,174 | 53,031,498 | 6,585,431 |
| 2014 | 92,429,636 | 67,705,892 | 15,335,036 | 53,464,326 | 6,693,727 |
| Per-capita treatment numbers | | | | | |
| 2004 | 0.220 | 0.005 | 0.645 | — | 0.286 |
| 2005 | 0.192 | 0.031 | 0.564 | 0.287 | 1.354 |
| 2006 | 0.272 | 0.053 | 0.832 | 0.779 | 2.405 |
| 2007 | 0.131 | 0.050 | 1.098 | 0.447 | 2.758 |
| 2008 | 0.127 | 0.039 | 0.581 | 0.367 | 4.743 |
| 2009 | 0.370 | 0.061 | 0.750 | 1.059 | 1.119 |
| 2010 | — | 0.040 | 1.267 | 0.516 | 0.821 |
| 2011 | 0.124 | 0.008 | 0.825 | 1.092 | 0.885 |
| 2012 | 0.213 | 0.005 | 2.842 | 1.038 | 1.612 |
| 2013 | 0.155 | 0.022 | 0.779 | 0.701 | 0.736 |
| 2014 | 0.115 | 0.029 | 0.773 | 0.526 | 0.748 |
| *Mean* | *0.192* | *0.031* | *0.996* | *0.681* | *1.588* |

Table S4: Prevalence estimates for countries in the Greater Mekong Subregion based on combined studies from the Malaria Atlas Project [14].

|  | **Years^a^** | **Number of studies** | **Total positive samples** | **Total number tested** | **Estimated prevalence** | **95% Confidence Interval** |
| --- | --- | --- | --- | --- | --- | --- |
| Viet Nam | 1994-1996 | 7 | 526 | 14,343 | 3.7% | (3.4–4.0%) |
| Thailand | 1993-1995 | 1 | 56 | 791 | 7.1% | (5.4–9.1%) |
| Cambodia | 1999-2001 | 3 | 762 | 12,663 | 6.0% | (5.6–6.4%) |
| Myanmar | 2001-2003 | 2 | 350 | 4,022 | 8.7% | (7.8–9.6%) |
| Lao People's Democratic Republic | 2001-2003 | 2 | 197 | 991 | 19.8% | (17.4–22.5%) |

^a^ To attempt to control for limited numbers of studies and sample sizes in the individual years that that correspond to the years that ACTs were introduced, all studies that were undertaken in a +/- 1 year window were pooled.

**Equations**

This section provides a mathematical description of the model used for the analysis.

*Population and compartments*

Define the compartments and indices as:

- S, susceptible; L, latent (liver-stage); I, infectious (gametocyte stage).
- Superscript 1 for wild-type strain; superscript 2 for K13 mutant strain.
- For the human model, subscript 1 for no immunity; subscript 2 for low immunity; subscript 3 for high immunity.

Therefore the total human population size (pop) and total mosquito population size $(\hat{pop})$ are given by

$pop=\sum_{j=1,2,3} \left( S_{j}+\sum_{i=1,2} L_{j}^{i}+I_{j}^{i} \right)$, $\hat{pop}=\hat{S}+\sum_{i=1,2} \hat{L}^{i}+\hat{I}^{i}=pop\times N/\hat{N}$ (see below for N, $\hat{N}$)

Deaths are assumed to occur with equal probability from each compartment and human / mosquito populations are held constant by assuming birth and death rates are equal. These are given by:

- $\mu=$ human birth rate into $S_{1}$ [1 / life expectancy]
- $\hat{\mu}=$ mosquito birth rate into $\hat{S}$ [1 / life expectancy]

*Transmission*

Transmission is defined in terms of bites per month, probability of transmission per bite, and the current infectious proportion of the population. Let

- N = bites per month (calibrated to human prevalence)
- $\hat{N}=$ bites per month per mosquito
- $\lambda_{MH}^{i}=$ prob transmission [M🡪H] per bite, strain i
- $\lambda_{HM}^{i}=$ prob transmission [H🡪M] per bite, strain i

Then the forces of infection for humans with wild-type $(\beta^{1})$, humans with mutants $(\beta^{2})$, mosquitoes with wild-type $(\hat{\beta}^{1})$ and mosquitoes with mutants $(\hat{\beta}^{2})$ are given by

$$\beta^{1}=N\lambda_{MH}^{1}\times\frac{\hat{I}^{1}}{\hat{pop}}$$

$$\beta^{2}=N\lambda_{MH}^{2}\times\frac{\hat{I}^{2}}{\hat{pop}}$$

$$\hat{\beta}^{1}=\hat{N}\lambda_{HM}^{1}\times\frac{I_{1}^{1}+\delta_{b1}^{1}I_{2}^{1}+\delta_{b1}^{1}I_{3}^{1}}{\mathrm{pop}}$$

$$\hat{\beta}^{2}=\hat{N}\lambda_{HM}^{2}\times\frac{I_{1}^{2}+\delta_{b1}^{2}I_{2}^{2}+\delta_{b1}^{2}I_{3}^{2}}{\mathrm{pop}}$$

*Transition rates*

Movements occur between compartments at the following rates:

- $l_{1}^{i},l_{2}^{i},l_{3}^{i}=$ 1 / average duration of latency [time from bite to infectious], strain i, with no, low or high immunity
- $\tau_{1}^{i},\tau_{2}^{i},\tau_{3}^{i}=$1 / average duration of infectiousness, strain i, with no, low or high immunity
- $\omega_{1}, \omega_{2}=$ 1 / average duration of low and high immunity

*Immunity*

Different immunity parameters are given by:

- $\delta_{a1}^{i},\delta_{a2}^{i}=$ reduction in M🡪H transmission of strain i with low or high immunity
- $\delta_{b1}^{i},\delta_{a2}^{i}=$ reduction in H🡪M transmission of strain i with low or high immunity
- $f$ = proportion of recovering humans with low immunity who develop high immunity

*Mutation*

The mutation rate within the human population was modelled to be proportional to the per capita treatment rate (average number of treatments per person per year in the population), the relative fitness of the mutant strain (the proportion of mutations that result in a person moving from a wild type classified strain to a mutant classified strain) and the ratio of mutant to wild-type infections. The mutation rate within mosquitoes was modelled to be proportional to the ratio of mutant to wild-type infections among mosquitoes. Specifically, define:

- $p_{fit}=$ the relative fitness of the mutant strain
- $T=$ the per capita treatment rate
- $m={max(1,\sum_{k=1,2,3} I_{k}^{2}}/{\sum_{k=1,2,3} I_{k}^{1}})$ = ratio of mutant to wild-type infections among humans
- $\hat{m}=max(1,{\hat{I}^{2}}/{\hat{I}^{1}})$ = ratio of mutant to wild-type infections among mosquitoes
- $\rho_{1},\rho_{2},\rho_{3}=$ rate of mutation in humans leading to co-infection, with no, low or high immunity (proportionality constants)
- $\hat{\rho}=$ rate of mutation in mosquitoes leading to co-infection (proportionality constant)

The per capita treatment rate $T$ was calculated as follows. For each level of immunity $k$, a proportion $\eta_{k}$ of infections were assumed to clear via treatment (as opposed to suppression within the host), where treatment has an efficacy of $\epsilon$. We require that $\eta_{1}>\eta_{2}>\eta_{3}$ since, for example, those with no clinical immunity are more likely to present with symptoms than those with partial immunity. Then the per capita treatment number is

$$T=\frac{\frac{1}{\epsilon}\sum_{k=1,2,3} \tau_{k}\eta_{k}\left( I_{k}^{1}+I_{k}^{2} \right)}{pop}$$

The values of $\eta_{1}, \eta_{2}, \eta_{3}$ were determined as part of the calibration process above to match $T$ to the settings in the Greater Mekong Subregion, ensuring that $\eta_{1}>\eta_{2}>\eta_{3}$.

*Drug pressure*

Drug pressure was implicitly included in the model by incorporation of the treatment term ($T$) in the within-host mutation rate. This mean that as the number of treatments distributed to a population increase, so does the relative rate of mutation emergence within individuals.

*Human population equations*

Note: superscript $i=1$ for wild type, $i=2$ for mutants

$$\frac{dS_{1}}{dt}=\mu\times\left( pop \right)-\left( \beta^{1}+\beta^{2} \right)S_{1}+\omega_{1}S_{2}-\mu S_{1}$$

$$\frac{dL_{1}^{i}}{dt}=\beta^{i}S_{1}-l_{1}^{i}L_{1}^{i}-\mu L_{1}^{i}$$

$$\frac{dI_{1}^{i}}{dt}=l_{1}^{i}L_{1}^{i}-\tau_{1}^{i}I_{1}^{i}+\left( -1 \right)^{i}\left( \rho_{1}p_{fit}mT+\beta^{2}p_{fit} \right)I_{1}^{1}-\mu I_{1}^{i}$$

$$\frac{dS_{2}}{dt}=\omega_{2}S_{3}-\omega_{1}S_{2}-\mu S_{2}+\sum_{i=1,2} \tau_{1}^{i}I_{1}^{i}+\left( 1-f \right)\tau_{2}^{i}I_{2}^{i}-\delta_{a1}^{i}\beta^{i}S_{2}$$

$$\frac{dL_{2}^{i}}{dt}={\delta_{a1}^{i}\beta}^{i}S_{2}-l_{2}^{i}L_{2}^{i}-\mu L_{2}^{i}$$

$$\frac{dI_{2}^{i}}{dt}=l_{2}^{i}L_{2}^{i}-\tau_{2}^{i}I_{2}^{i}+\left( -1 \right)^{i}\left( \rho_{2}p_{fit}mT+\beta^{2}p_{fit} \right)I_{2}^{1}-\mu I_{2}^{i}$$

$$\frac{dS_{3}}{dt}={-\omega}_{2}S_{3}-\mu S_{2}+\sum_{i=1,2} {f\tau}_{2}^{i}I_{2}^{i}+\tau_{3}^{i}I_{3}^{i}-\delta_{a2}^{i}\beta^{i}S_{3}$$

$$\frac{dL_{3}^{i}}{dt}={\delta_{a2}^{i}\beta}^{i}S_{3}-l_{3}^{i}L_{3}^{i}-\mu L_{3}^{i}$$

$$\frac{dI_{3}^{i}}{dt}=l_{3}^{i}L_{3}^{i}-\tau_{3}^{i}I_{3}^{i}+\left( -1 \right)^{i}\left( \rho_{3}p_{fit}mT+\beta^{2}p_{fit} \right)I_{3}^{1}-\mu I_{3}^{i}$$

*Mosquito population equations*

$$\frac{d\hat{S}}{dt}=\mu\times\left( pop \right)-\left( \hat{\beta}^{1}+\hat{\beta}^{2} \right)\hat{S}-\mu\hat{S}$$

$$\frac{d\hat{L}^{i}}{dt}=\hat{\beta}^{i}\hat{S}-\hat{l}\hat{L}^{i}-\mu\hat{L}^{i}$$

$\frac{d\hat{I}^{i}}{dt}=\hat{l}\hat{L}^{i}+\left( -1 \right)^{i}\hat{\rho}\hat{m}\hat{I}^{1}-\mu\hat{I}^{i}$

**Additional plots**

*Example model simulation*


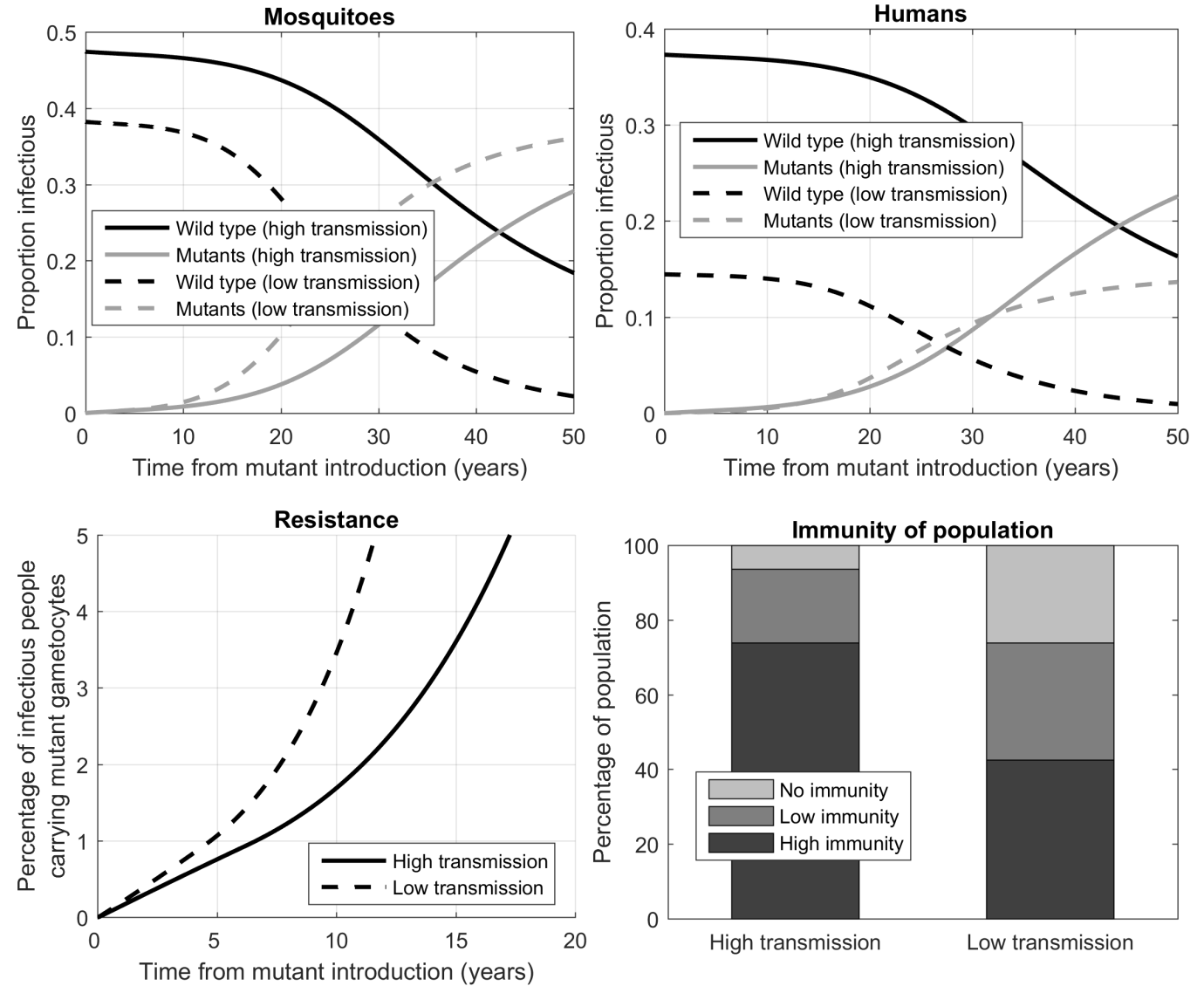


**Figure S2: Modelled emergence of mutant strains in two different prevalence settings.** After its introduction, the prevalence of the mutant strain (strain with K13 mutation that confers a slow clearing phenotype) increases among both mosquitoes (top-left) and humans (top-right), as the prevalence of wild-type decreases. In high prevalence settings, a greater proportion of people have high levels of immunity than in low prevalence settings (bottom-right), and as a proportion of all infections among humans, confirmed partial artemisinin resistance is predicted to take longer to detect (bottom-left).

*Relationship between prevalence and incidence in the model*

The modelled relationship is similar to what has been derived recently by Cameron et al. [36].


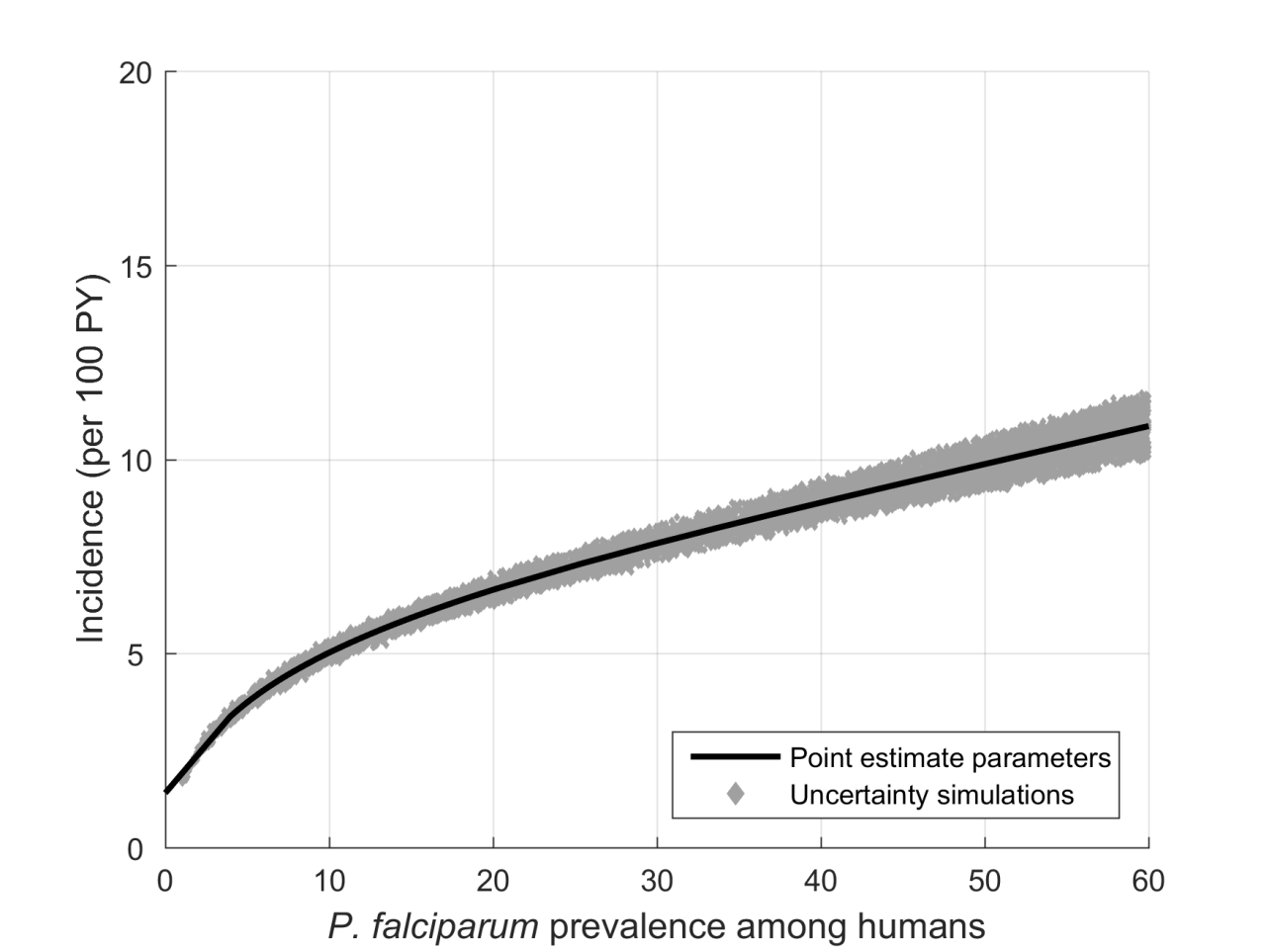


**Figure S3: Modelled relationship between prevalence and incidence (per 100 person years [PY]).**

**References**

1. Walker PG, Griffin JT, Ferguson NM, Ghani AC: **Estimating the most efficient allocation of interventions to achieve reductions in Plasmodium falciparum malaria burden and transmission in Africa: a modelling study**. *The Lancet Global Health* 2016.

2. World Health Organization: **Status report in artemisinin and ACT resistance**. *Geneve* 2015.

3. Koella J, Antia R: **Epidemiological models for the spread of anti-malarial resistance**. *Malaria Journal* 2003, **2**(1):3.

4. Koella JC: **On the use of mathematical models of malaria transmission**. *Acta tropica* 1991, **49**(1):1-25.

5. Aron JL: **Mathematical modelling of immunity to malaria**. *Mathematical Biosciences* 1988, **90**(1):385-396.

6. Chitnis N, Cushing J, Hyman J: **Bifurcation analysis of a mathematical model for malaria transmission**. *SIAM Journal on Applied Mathematics* 2006, **67**(1):24-45.

7. Labadin J, Kon C, Juan S: **Deterministic malaria transmission model with acquired immunity**. In: *Proceedings of the World Congress on Engineering and Computer Science: 2009*; 2009: 20-22.

8. Tumwiine J, Mugisha J, Luboobi L: **A mathematical model for the dynamics of malaria in a human host and mosquito vector with temporary immunity**. *Applied Mathematics and Computation* 2007, **189**(2):1953-1965.

9. O'Meara WP, Smith DL, McKenzie FE: **Potential impact of intermittent preventive treatment (IPT) on spread of drug-resistant malaria**. *PLoS Med* 2006, **3**(5):e141.

10. Ataide R, Ashley EA, Powell R, Chan J-A, Malloy MJ, O’Flaherty K, Takashima E, Langer C, Tsuboi T, Dondorp AM *et al*: **Host immunity to Plasmodium falciparum and the assessment of emerging artemisinin resistance in a multinational cohort**. *Proceedings of the National Academy of Sciences* 2017.

11. Kennedy J: **Particle swarm optimization**. In: *Encyclopedia of machine learning.* edn.: Springer; 2011: 760-766.

12. World Health Organization: **Emergency response to artemisinin resistance in the greater Mekong subregion**. *Regional framework for action* 2013, **2015**.

13. Hay SI, Snow RW: **The Malaria Atlas Project: developing global maps of malaria risk**. *PLoS Med* 2006, **3**(12):e473.

14. Malaria Atlas Project: [**http://www.map.ox.ac.uk/**](http://www.map.ox.ac.uk/). 2016.

15. UNICEF.: **Malaria and children: progress in intervention coverage.** . *UNICEF; New York* 2007, **ISBN 978-92-806-4184-4**.

16. Dalrymple DG: **Artemisia annua, artemisinin, ACTs and malaria control in Africa: tradition, science and public policy**: DG Dalrymple; 2013.

17. White N: **Antimalarial drug resistance and combination chemotherapy**. *Philosophical Transactions of the Royal Society of London B: Biological Sciences* 1999, **354**(1384):739-749.

18. World Health Organization: **Global Report on Antimalarial Drug Efficacy and Drug Resistance: 2000-2010. Available from** [**http://apps.who.int/iris/handle/10665/44449**](http://apps.who.int/iris/handle/10665/44449). 2010.

19. Anderson RM, May RM, Anderson B: **Infectious diseases of humans: dynamics and control**, vol. 28: Wiley Online Library; 1992.

20. Chitnis N, Hyman JM, Cushing JM: **Determining important parameters in the spread of malaria through the sensitivity analysis of a mathematical model**. *Bulletin of mathematical biology* 2008, **70**(5):1272-1296.

21. Tripura R, Peto TJ, Chalk J, Lee SJ, Sirithiranont P, Nguon C, Dhorda M, Seidlein L, Maude RJ, Day NP: **Persistent Plasmodium falciparum and Plasmodium vivax infections in a western Cambodian population: implications for prevention, treatment and elimination strategies**. *Malaria journal* 2016, **15**(1):181.

22. Griffin JT, Ferguson NM, Ghani AC: **Estimates of the changing age-burden of Plasmodium falciparum malaria disease in sub-Saharan Africa**. *Nature communications* 2014, **5**.

23. White MT, Verity R, Griffin JT, Asante KP, Owusu-Agyei S, Greenwood B, Drakeley C, Gesase S, Lusingu J, Ansong D *et al*: **Immunogenicity of the RTS,S/AS01 malaria vaccine and implications for duration of vaccine efficacy: secondary analysis of data from a phase 3 randomised controlled trial**. *Lancet Infect Dis* 2015, **15**(12):1450-1458.

24. John CC, Moormann AM, Pregibon DC, Sumba PO, McHugh MM, Narum DL, Lanar DE, Schluchter MD, Kazura JW: **Correlation of high levels of antibodies to multiple pre-erythrocytic Plasmodium falciparum antigens and protection from infection**. *Am J Trop Med Hyg* 2005, **73**(1):222-228.

25. Bousema T, Roeffen W, Meijerink H, Mwerinde H, Mwakalinga S, van Gemert GJ, van de Vegte-Bolmer M, Mosha F, Targett G, Riley EM *et al*: **The dynamics of naturally acquired immune responses to Plasmodium falciparum sexual stage antigens Pfs230 & Pfs48/45 in a low endemic area in Tanzania**. *PLoS One* 2010, **5**(11):e14114.

26. Churcher TS, Trape JF, Cohuet A: **Human-to-mosquito transmission efficiency increases as malaria is controlled**. *Nat Commun* 2015, **6**:6054.

27. Clements A, Paterson G: **The analysis of mortality and survival rates in wild populations of mosquitoes**. *Journal of applied ecology* 1981:373-399.

28. Mandal S, Sarkar RR, Sinha S: **Mathematical models of malaria-a review**. *Malar J* 2011, **10**(202):10.1186.

29. Vaughan JA: **Population dynamics of Plasmodium sporogony**. *Trends in parasitology* 2007, **23**(2):63-70.

30. Baton LA, Ranford-Cartwright LC: **Spreading the seeds of million-murdering death: metamorphoses of malaria in the mosquito**. *Trends in parasitology* 2005, **21**(12):573-580.

31. Charlwood J, Smith T, Billingsley P, Takken W, Lyimo E, Meuwissen J: **Survival and infection probabilities of anthropophagic anophelines from an area of high prevalence of Plasmodium falciparum in humans**. *Bulletin of Entomological Research* 1997, **87**(05):445-453.

32. Charlwood J, Graves P, Birley M: **Capture-recapture studies with mosquitoes of the group of Anopheles punctulatus Dönitz (Diptera: Culicidae) from Papua New Guinea**. *Bulletin of entomological research* 1986, **76**(2):211-227.

33. Killeen GF, McKENZIE FE, Foy BD, Schieffelin C, Billingsley PF, Beier JC: **A simplified model for predicting malaria entomologic inoculation rates based on entomologic and parasitologic parameters relevant to control**. *The American journal of tropical medicine and hygiene* 2000, **62**(5):535-544.

34. Mehlhorn H, Armstrong PM: **Encyclopedic reference of parasitology: Diseases, treatment, therapy**, vol. 2: Springer Science & Business Media; 2001.

35. Dietz K, Molineaux L, Thomas A: **A malaria model tested in the African savannah**. *Bulletin of the World Health Organization* 1974, **50**(3-4):347.

36. Cameron E, Battle KE, Bhatt S, Weiss DJ, Bisanzio D, Mappin B, Dalrymple U, Hay SI, Smith DL, Griffin JT: **Defining the relationship between infection prevalence and clinical incidence of Plasmodium falciparum malaria**. *Nature communications* 2015, **6**.
